# Client Choice May Provide an Economic Incentive for Veterinary Practices to Invest in Sustainable Infrastructure and Climate Change Education

**DOI:** 10.3389/fvets.2020.622199

**Published:** 2021-01-18

**Authors:** Sarah B. Deluty, Danielle M. Scott, Sabrina C. Waugh, Veronica K. Martin, Katherine A. McCaw, Jessica R. Rupert, Tracy L. Webb, Stacey A. Baumgarn, Molly J. Carpenter, Colleen G. Duncan

**Affiliations:** ^1^College of Veterinary Medicine and Biomedical Sciences, Fort Collins, CO, United States; ^2^Department of Clinical Sciences, College of Veterinary Medicine and Biomedical Sciences, Fort Collins, CO, United States; ^3^Facilities Management, Fort Collins, CO, United States; ^4^Department of Microbiology, Immunology and Pathology, College of Veterinary Medicine and Biomedical Sciences, Fort Collins, CO, United States

**Keywords:** climate–change, veterinary, economics, client, animal, pet, sustainability, business

## Abstract

**Objective:** To assess how pet owners perceive the role of veterinary medicine in addressing climate change and animal health and determine if there is a client-driven economic incentive to establish sustainable veterinary business practices.

**Sample:** 1,044 dog and/or cat owners residing in the United States who had used veterinary services within the last 3 years.

**Procedures:** An online Amazon mTurk survey about climate change and the perceived effects on client-owned dogs and cats was distributed to pet owners.

**Results:** Most respondents believe climate change is occurring, and two-thirds of pet owners would value knowing their veterinarian received training on the animal health impacts of climate change. Over half of the respondents would pay more for veterinary services at a clinic with a reduced environmental impact. Additionally, clients would value some form of sustainability certification to aid in identification of such practices. Demographic influences found to be statistically significant included age, political ideology and where one resides (i.e., urban, suburban, or rural) whereas gender and income level, were not found to be significant.

**Conclusions and Clinical Relevance:** Our data suggest there is an economic incentive for veterinary professionals to be knowledgeable about the health impacts of climate change and to implement and market sustainable practice initiatives. Prioritizing sustainable practice initiatives and climate change education in veterinary practices has the potential to mutually benefit both practitioner and client through shared patient health and financial incentives.

## Introduction

Veterinary professionals play a unique role in public health, prevention of zoonoses, and food safety and security. Therefore, veterinarians have been integral in the establishment of the One Health concept which “recognizes that the health of people is closely connected to the health of animals and our shared environment” (1). Despite appreciating the environmental influence on health, the veterinary profession has been criticized for its lack of action on issues related to climate change (2), which the public health community has identified as “the biggest global health threat of the 21st century” (3). The human medical field is responding to climate challenges by incorporating related curriculum into medical school programs and continuing education and developing methods and policies aimed at reducing the environmental impact of hospitals (4–6). These efforts are appreciated by clients as demonstrated by increased satisfaction and likelihood to return to a practice (7). Unfortunately, similar measures have not been widely implemented in the veterinary field.

Recent surveys of veterinary students (8) and veterinarians (9) identified significant gaps in education regarding climate change and animal health, even though the overwhelming majority believe this education is important to their careers. Both veterinarians and veterinary students feel that climate change is important to animal health and that practitioners should play a leadership role in promoting environmental sustainability within offices, clinics, and hospitals (8, 9). Given this strong conviction in both practicing and future veterinarians, it is unknown why animal health professionals are less engaged in sustainability activities than their human counterparts.

Perceived barriers to implementing business practices that reduce environmental impact are often financial (10). However, these costs can be offset by increased consumer concern about the environment and subsequent redirection of investment in sustainable businesses. Nearly two-thirds of consumers feel it's their responsibility to make sustainable purchases (11). Moreover, 63% of Americans are hopeful businesses will take the lead on environmental issues, with 87% reporting they would purchase from a business based on company values (12). Finally, from 2006 to 2010, the top 100 sustainable consumer goods companies around the globe had significantly higher mean sales growth, return on assets, profits before taxation, and cash flow from some sectors when compared to control companies (11).

Similar to consumer goods, veterinary client spending and satisfaction is a driver of the market (13, 14). However, no previous studies have assessed the expectations of veterinary clients regarding their interest in environmental sustainability at clinics and hospitals. The objective of this study was to investigate veterinary clients' views on the perceived health effects of climate change on their companion animals as well as their expectations of veterinarians' ability to educate clients on the health impacts of climate change on pets. We hypothesized that economic drivers for sustainable practices present in other consumer markets would also apply to veterinary medicine. If so, client expectation and demand will be another incentive, in addition to the veterinary profession's commitment to public health, for veterinarians to incorporate environmentally sustainable practices.

## Materials and Methods

An online survey was conducted using Qualtrics^1^ software and disseminated via Amazon's Mechanical Turk (mTurk)^2^, which pays workers to complete HITs (Human Intelligence Tasks) through a series of questions. Questions were developed by the research team and piloted with pet owners. Some questions similar to those used by Pollard et al. (2020) in a survey of veterinary students were included to facilitate comparison of responses about climate change and education with veterinary clients. A full list of questions is available in the Supplementary Material. Respondents were required to be 18 years or older, live in the United States, and own at least one cat and/or dog who had received veterinary services within the last 3 years. The survey was approved by the Colorado State University Institutional Review Board prior to distribution. The mTurk request was available October 31–November 10, 2019.

The questionnaire consisted of one opinion/free, two Likert scale, three select-all-that-apply, and 10 multiple choice responses. Two screening questions were used to validate respondents and inclusion criteria. The first screening question confirmed the respondent owned a pet they had taken to a veterinarian in the last 3 years, and the second required a correct response to detect artificial accounts (see Supplementary Material). None of the remaining questions required a response to submit the survey. Participants provided their MTurk ID number to be compensated ($1) for completing the survey but responses were otherwise anonymous. Descriptive and comparative (Pearson's chi-squared tests) data analysis was conducted using commercially available software^3^,^4^. As not all questions were answered by each survey respondent, percentages were calculated out of total responses per question.

## Results

### Demographics of Respondents

1,068 responses were received. Responses that failed to answer the screening questions correctly (*n* = 24) were excluded, resulting in 1,044 respondents for the final analysis. Of these, 46.1% (481/1,044) of respondents had one dog or cat at home that received veterinary care in the past 3 years. 34.8% (363/1,044) had two dogs or cats, 11.1% (116/1044) had three dogs or cats, and 8.0% (84/1,044) of respondents had four or more dogs or cats.

54.2% (566/1,044) of respondents identified as female and 45.1% (471/1,044) as male, with the remaining 0.7% (7/1,044) preferring not to answer. The majority of the respondents were aged 25–34 years old (32.3%, 337/1,044), followed by 35–44 years old (28.9%, 302/1,044), 45–54 years (15.8%, 165/1,044), 55–64 years (12.2%, 127/1,044), 18–24 years (6.1%, 64/1,044), and 65 years or older (4.7%, 49/1,044). Annual household income was reported as 13.2% (138/1,044) making < $24,999, 28.3% (295/1,044) making $25,000–$49,999, 26.5% (277/1,044) making $50,000–$74,999, 16.5% (172/1,044) making $75,000–$99,999, 10.3% (108/1,044) making $100,000–$149,999, and 5.2% (54/1,044) making over $150,000.

Responses were received from every state in the United States except Wyoming. Most respondents reported living in a suburban area (55.8%, 583/1,044), followed by an urban area (29.7%, 310/1,044), with the least respondents reporting living in a rural area (14.5%, 151/1,044). Regarding political affiliation, 30.5% (318/1,044) of respondents identified as somewhat liberal, followed by 21.6% (225/1,044) who identified as very liberal, 19.7% (206/1,044) as moderate, 17.9% (187/1,044) as somewhat conservative, and 10.1% (105/1,044) of respondents considered themselves very conservative. The remaining three respondents (0.3%, 3/1,044) identified as “other.”

In a typical year, the majority (38.4%, 401/1,044) of pet owners reported spending between $100 and $299 on veterinary care, followed by 31.5% (329/1,044) who spent between $300 and $599. Of the remaining respondents, 11.4% reported spending $99 (119/1,044) or less, 10.8% (113/1,044) spent $600–$999, and 7.9% (82/1,044) spent $1,000 or more.

### Client Perspectives on Climate Change and Their Pets

**Figure 1:**
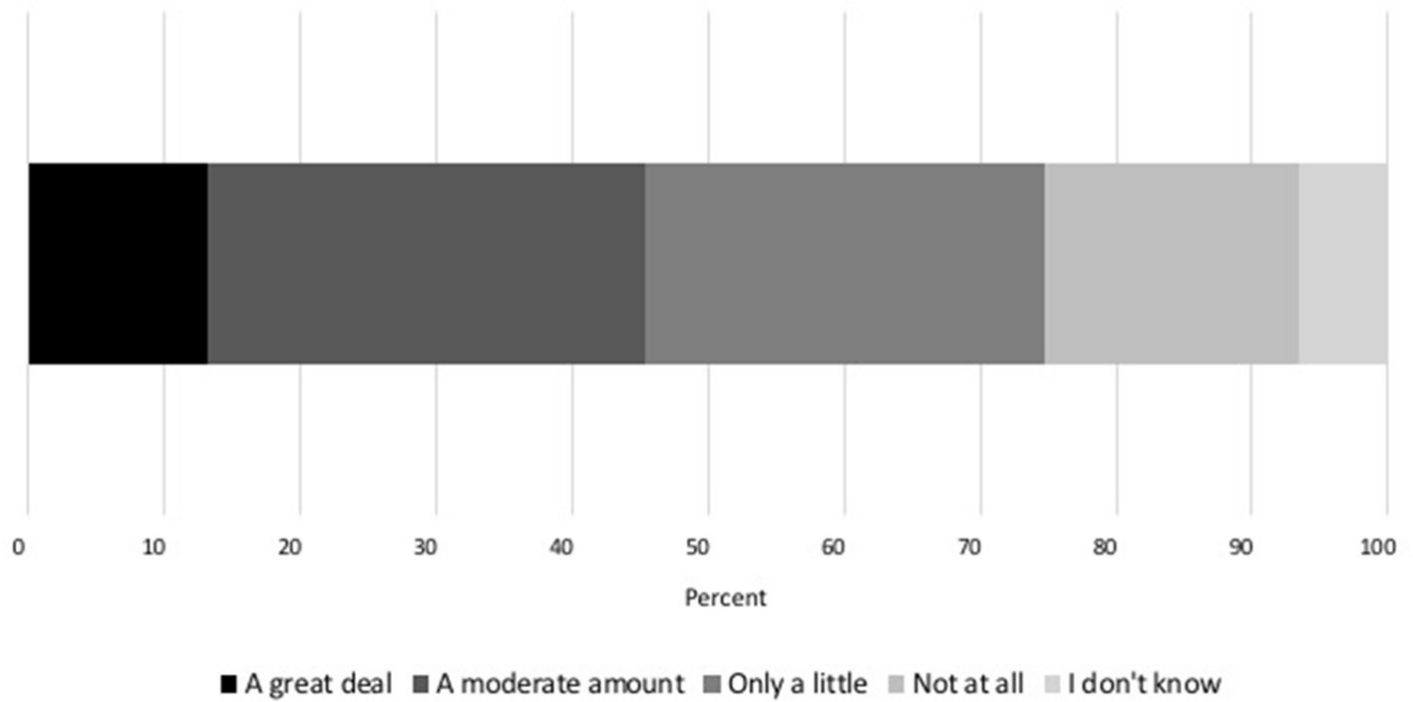
Percentage of respondents who believe, or do not believe, that climate change is relevant to their pet's health, and the degree of their belief.

The overwhelming majority of respondents (84.9%, 886/1,044) reported believing that climate change is occurring, while 7.0% (73/1,044) did not believe climate change is occurring and 8.1% (85/1,044) did not know if climate change is occurring or not. When asked how much, if at all, climate change is relevant to their pet's health, 74.7% (780/1,044) of pet owners responded it has at least some relevance (Figure 1; Table 1). Excluding respondents who replied that they did not know, the frequency of those believing climate change is relevant to animal health (to any degree) compared to those that did not varied significantly by political affiliation (χ^2^ (5, *n* = 975) = 126.27, *p* < 0.001) and age (χ^2^ (5, *n* = 975) = 12.24, *p* = 0.03); there was not a significant difference in gender, income, or region of residence.

**Table 1:** Percentages of respondents by demographics indicating if climate change is relevant to their pet's health.

**Demographic Factor**	**Yes**	**No**	**I don't know**
**Region**			
Urban	78% (241/310)	15% (46/310)	7% (23/310)
Suburban	74% (433/583)	20% (115/583)	6% (35/583)
Rural	70% (106/151)	23% (34/151)	7% (23/310)
**Income**			
$200,000+	79% (22/28)	11% (3/28)	11% (3/28)
$150,000–199,999	69% (18/26)	27% (7/26)	4% (1/26)
$100,000–149,999	66% (71/108)	25% (27/108)	9% (10/108)
$75,000–99,999	78% (135/172)	13% (23/172)	8% (14/172)
$50,000–74,999	77% (213/277)	18% (50/277)	5% (14/277)
$25,000–49,999	77% (227/295)	19% (55/295)	4% (13/295)
<$24,999	68% (94/138)	22% (30/138)	10% (14/138)
Politics^*^			
Very Conservative	49% (51/105)	48% (50/105)	4% (4/105)
Somewhat Conservative	62% (115/187)	32% (60/187)	6% (12/187)
Moderate	69% (142/206)	20% (41/206)	11% (23/206)
Somewhat Liberal	84% (267/318)	11% (35/318)	5% (16/318)
Very Liberal	90% (203/225)	4% (8/225)	6% (14/225)
Other	67% (2/3)	33% (1/3)	0% (0/3)
Age^*^			
18–24	78% (50/64)	11% (7/64)	11% (7/64)
25–34	81% (272/337)	15% (51/337)	4% (14/337)
35–44	73% (219/302)	19% (56/302)	9% (27/302)
45–54	71% (117/165)	23% (38/165)	6% (10/165)
55–64	71% (90/127)	23% (29/127)	6% (8/127)
65+	65% (32/49)	29% (14/49)	6% (3/49)
**Gender**			
Female	76% (429/566)	16% (92/566)	8% (45/566)
Male	73% (345/471)	22% (103/471)	5% (23/471)
Prefer not to answer or gender not listed	86% (6/7)	0% (0/7)	14% (1/7)

* *Statistically significant at p < 0.05*.

**Figure 2:**
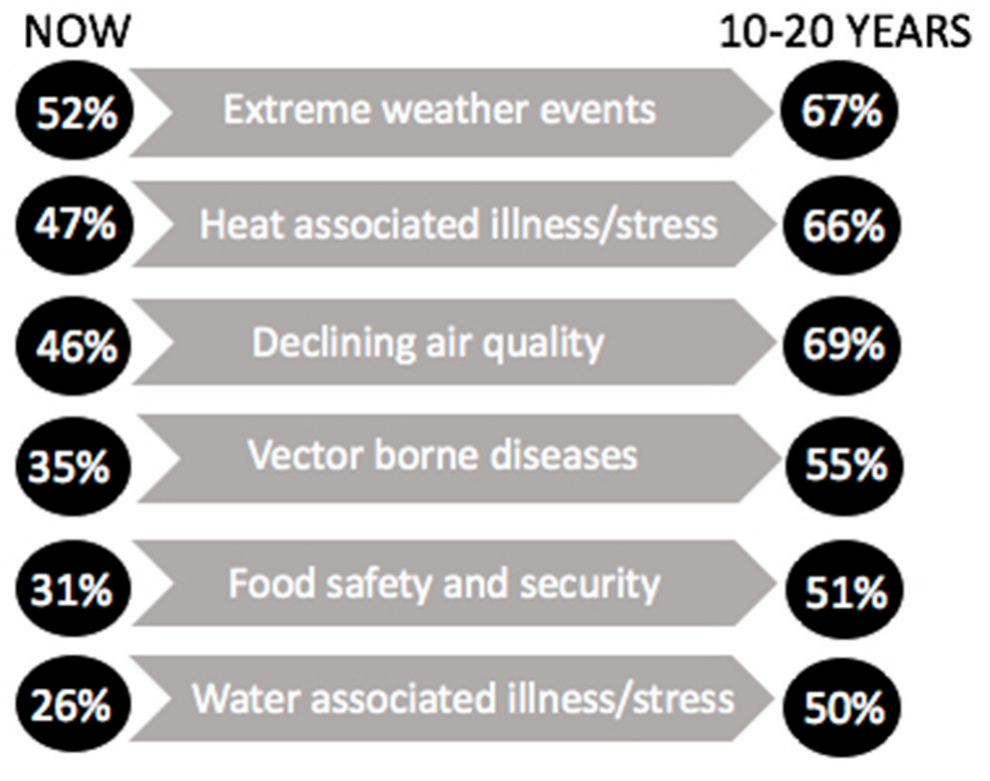
Percentage of respondents indicating how climate change is currently affecting their pet's health and how they might be affected in the next 10–20 years.

Agreement with the ways climate change is currently affecting pet health (extreme weather events, heat associated illness and stress, declining air quality, vector borne disease transmission, reduced food security, quality and biosecurity, water associated illness or stress), and how it might in the next 10–20 years, is summarized in (Figure 2). Notably, all categories showed a 15% or greater increase from today's perceived effects to what respondents expected in the next 10–20 years.

Respondents were provided the opportunity to contribute free text responses regarding their concerns about climate change and their pets. The most common write-in concerns were about the safety of outdoor spaces for exercise with their pets (*n* = 24), food scarcity and increased food costs (*n* = 19), exposure to allergens (*n* = 13), increased stress or behavioral changes in their pets in response to environmental or owner stress (*n* = 11), chemicals and toxins in the environment (*n* = 10), increased human movement resulting in more relinquished pets (*n* = 9), changing wildlife behaviors resulting in increased pet exposure to diseases and predation (*n* = 7), and reduced life expectancy of their pets (*n* = 6).

### Client Perspectives on Climate Change and Their Veterinarian

**Figure 3:**
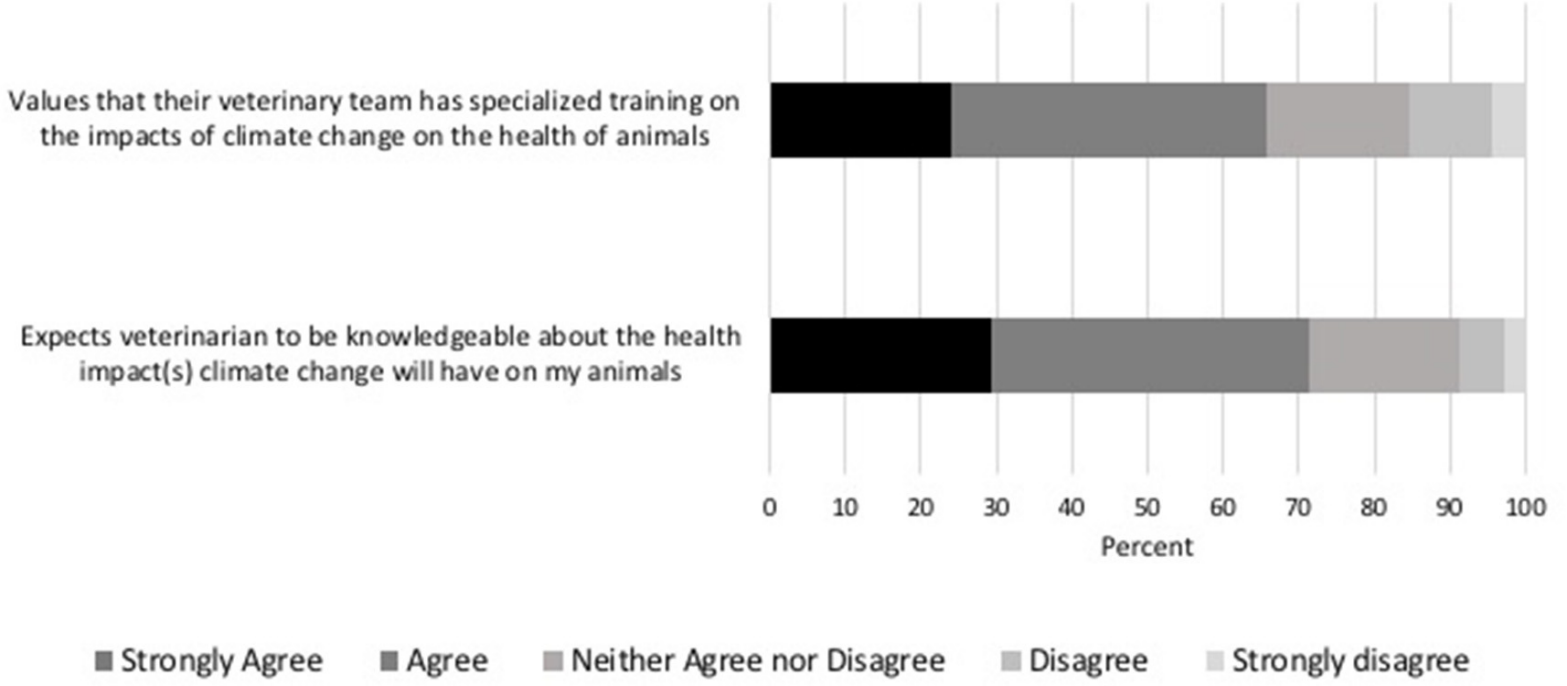
Percentage of respondents indicating their level of agreement on the statements, “I would value knowing that my veterinary team had undergone specialized training on the impacts of climate change on the health of animals,” and “I would like my vet to be knowledgeable about the health impact(s) climate change will have on my animals”.

There was an overall expectation of veterinarians to be knowledgeable about the health impacts of climate change on animals and value ascribed to those with formal training on the topic. Most respondents (71.4%, 746/1,044) expected their veterinarian to be knowledgeable about the impacts of climate change on their pet's health, 8.6% (90/1,044) did not expect their veterinarian to be knowledgeable, and 19.9% (208/1,044) had no expectation. Training on the impacts of climate change on the health of animals by the veterinary team was valued by 65.6% (685/1,044) of respondents, while 15.5% (162/1,044) did not value training in this area, and 18.9% (197/1,044) did not have an opinion (Figure 3).

**Figure 4:**
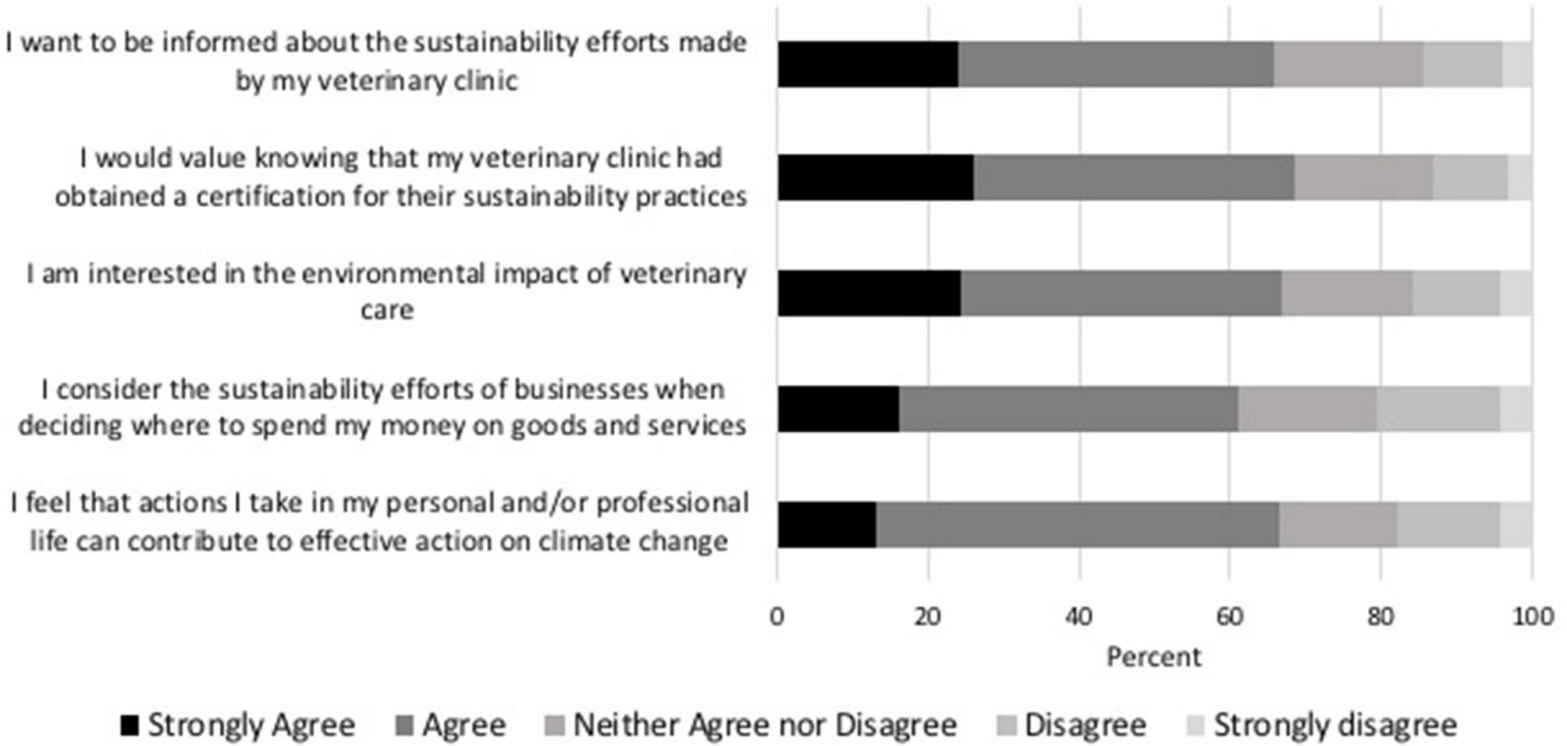
Percentage of respondents indicating their level of agreement with potential values and expectations regarding veterinary clinics and sustainable business practices.

Respondents' perceptions on sustainability efforts of veterinary practices and the environmental impact of veterinary care are presented in (Figure 4). Most respondents (65.8%, 687/1,044) would like to be informed about the efforts their veterinary clinic makes to reduce environmental impact, would value a certification for these sustainability efforts (68.6%, 717/1,044), and were interested in the current environmental impacts of veterinary care (66.8%, 697/1,044). Most respondents (60.9%, 636/1,044) also reported that they considered sustainability before making purchases and believed that their personal and professional decisions can contribute to effective action on climate change (66.5%, 694/1,044).

**Figure 5:**
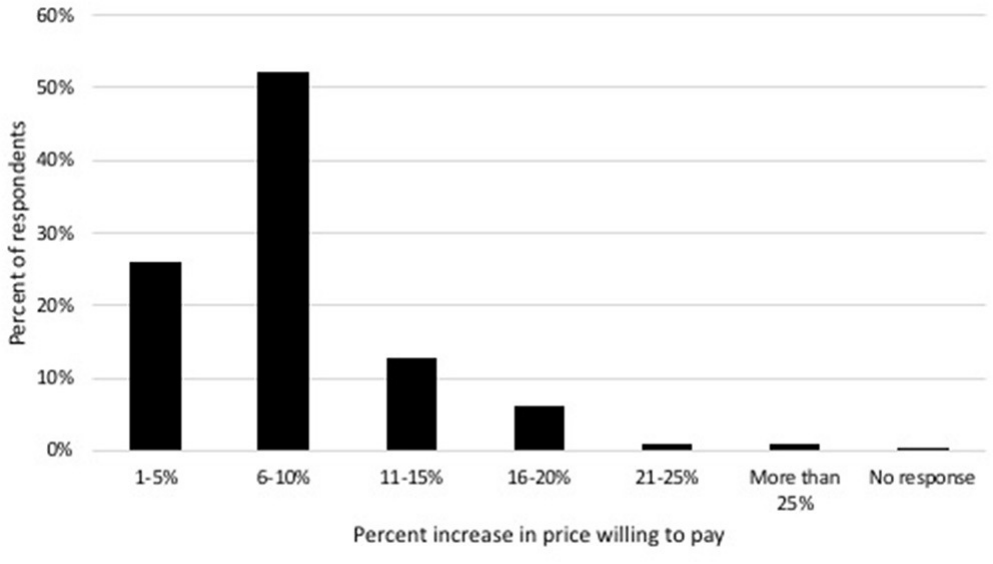
Breakdown of how much more clients are willing to pay for sustainable business practices of those who indicated they are willing to pay more.

The majority (55.8%, 583/1,044) of respondents stated that they would be willing to pay more money for veterinary services at a clinic that has significantly reduced their environmental impact (Figure 5). This willingness to pay more varied significantly by age [χ^2^ (5, *n* = 1,044) = 19.62, *p* = 0.001], neighborhood type [i.e., urban, suburban, or rural; χ^2^ (2, *n* = 1,044) = 8.67, *p* = 0.013], and political affiliation [χ^2^ (5, *n* = 1,044) = 105.62, *p* < 0.001] but was not influenced by respondent gender or household income (Table 2).

**Table 2:** Percentages of respondents by demographics responding to the question: Would you be willing to pay more money for veterinary services at a clinic that has significantly reduced their environmental impact?

**Demographic Factor**	**Yes**	**No**
Region^*^		
Urban	62% (193/310)	38% (117/310)
Suburban	54% (316/583)	46% (267/583)
Rural	49% (74/151)	51% (77/151)
**Income**		
$200,000+	50% (14/28)	50% (14/28)
$150,000–199,999	46% (12/26)	54% (14/26)
$100,000–149,999	58% (63/108)	42% (45/108)
$75,000–99,999	59% (102/172)	41% (70/172)
$50,000–74,999	58% (162/277)	42% (115/277)
$25,000–49,999	55% (162/295)	45% (133/295)
<$24,999	49% (68/138)	51% (70/138)
Politics^*^		
Very Conservative	29% (30/105)	71% (75/105)
Somewhat Conservative	36% (67/187)	64% (120/187)
Moderate	54% (112/206)	46% (94/206)
Somewhat Liberal	64% (204/318)	36% (114/318)
Very Liberal	75% (169/225)	25% (56/225)
Other	33% (1/3)	67% (2/3)
Age^*^		
18–24	66% (42/64)	34% (22/64)
25–34	63% (213/337)	37% (124/337)
35–44	53% (16/30)	47% (14/30)
45–54	51% (84/165)	49% (81/165)
55–64	50% (64/127)	50% (63/127)
65+	39% (19/49)	61% (30/49)
**Gender**		
Female	57% (320/566)	43% (246/566)
Male	55% (257/471)	45% (214/471)
Prefer not to answer or gender not listed	86% (6/7)	14% (1/7)

* *Statistically significant*.

**Figure 6:**
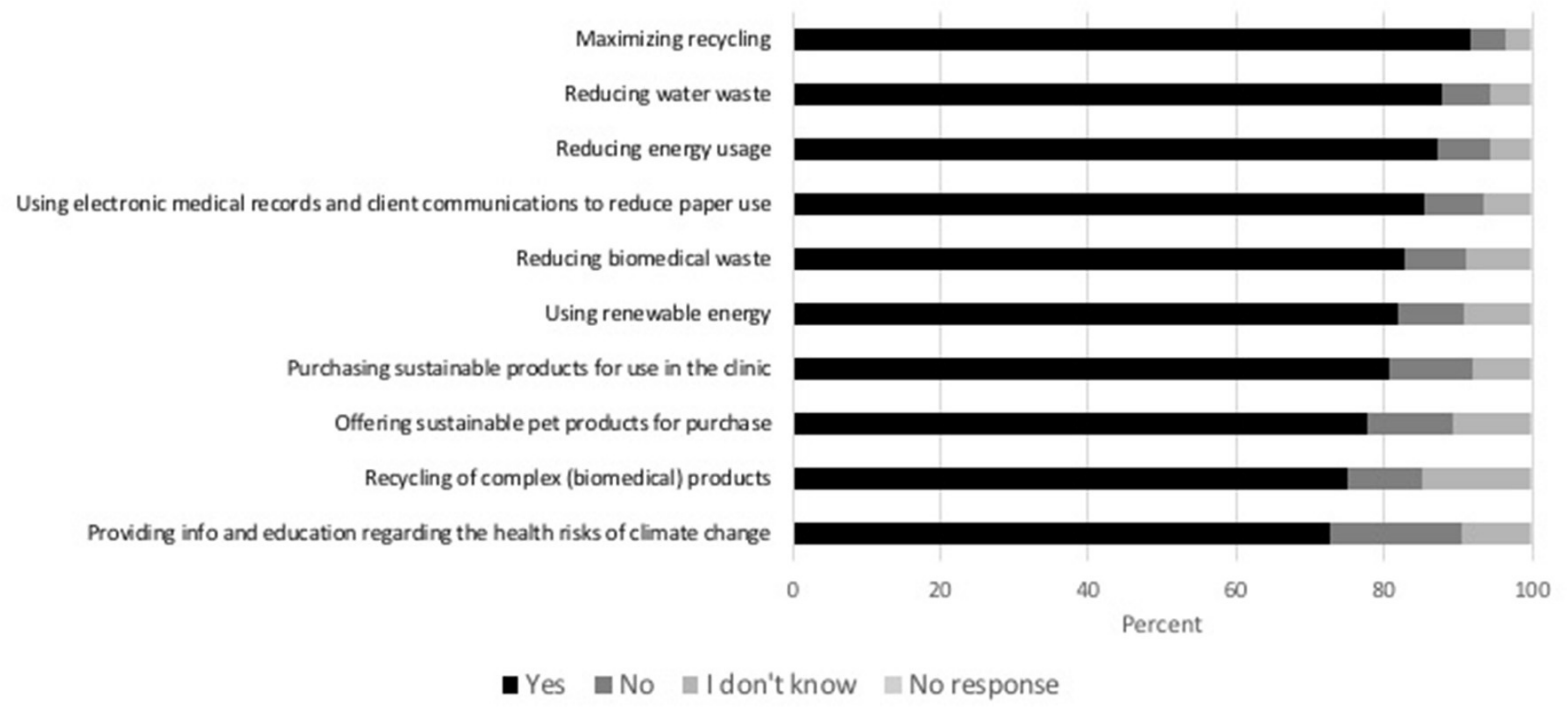
Percent of clients indicating the measures they would like to see veterinary clinics take to address the issue of climate change.

Respondents were asked how they would like to see veterinary clinics help address the issue of climate change. Greater than 70% of all respondents wanted to see veterinary clinics take the following actions: recycling (91.7%, 957/1,044), complex product recycling (75.0%, 783/1,044), reduction of water use (87.8%, 917/1,044), biomedical waste (82.8%, 864/1,044), reduction in energy use (87.2%, 910/1,044), use of paperless medical records (85.3%, 891/1,044), increase in renewable energy (81.8%, 854/1,044), sustainable product purchasing (80.7%, 843/1,044), sustainable products offered to consumers (77.7%, 811/1,044), and client education regarding the health risks of climate change (72.7%, 759/1,044) (Figure 6).

## Discussion

Results from this study contribute to the growing body of evidence that veterinarians have both personal and financial support from their clients to become better advocates and actors to combat climate change. Over 85% of responding dog and cat owners believe climate change is occurring, which is notably higher than the national average of 71% (15). Most clients surveyed believe that climate change influences the health of their pet and expect their veterinarians to be educated on this topic. Unfortunately this education is not widely available in the veterinary field, as recent surveys reported more than 75% of veterinary students and 90% of practicing veterinarians did not have or were unaware of resources available to learn more about the effects of climate change on animal health (8, 9). This highlights a significant gap in knowledge, training, or access to educational resources that does not meet the needs or expectations of veterinary health professionals or their clients.

Most clients reported that they are willing to pay more money for service from a “sustainable clinic.” Of those who would pay more, more than half of respondents would be willing to pay up to 6–10% more. A previous study identified that 74% of veterinary clients would continue to use their veterinarian if prices were raised 10%, however that percentage dropped to 58% when prices were raised 20% (13). Harvard Business Review reported that consumer goods companies can charge up to 20% price premiums and increase revenue up to 20% based on positive corporate responsibility performance (11). Veterinary practices have reported robust competition for clients (13), and our results suggest there is a strong economic argument for clinics to consider environmental sustainability in practice development plans. As many consumers consider the efforts made by businesses to reduce their environmental impact before deciding where to spend their money, implementation of marketing or certifications would assist veterinary clients in practice selection. Our study also indicated that clients value efforts to reduce environmental impact that are in-line with those that the American Veterinary Medical Association (AVMA) already recommends (16); these guidelines could serve as a resource to begin implementing such changes.

Political differences are known to influence environmental attitudes and can present a challenge in discussions regarding climate change (17–19). Not surprisingly political orientation was also a significant factor influencing pet owners' belief that climate change influences animal health and their willingness to pay more for veterinary services that have a reduced environmental impact. While pet owners who identified as liberal were statistically more concerned about the topic, more than 60% of those identifying as conservative still believed climate change is happening. Veterinary students and veterinarians have previously reported that the most common barrier to discussing climate change topics is perceived potential for damage to the veterinary-client relationship due to political discordance (8, 9). However, results of our study suggest that perceived political affiliation should not deter veterinarians from addressing associated health risks. Age was also found to be a statistically significant factor as over 70% of respondents under the age of 64 believed that climate change is relevant to pet health; the percentage decreased to 65% for respondents 65 and older. Collectively this information reiterates the need for animal health professionals to receive education on climate change which includes language and methods to discuss climate change as a health issue to facilitate conversations across political and generational divides (4, 20, 21).

This study utilized mTurk, a crowdsourcing website, to disseminate the survey and collect data. Studies of the online platform mTurk have found that the respondents come from more varied demographics as compared to both traditional and other internet survey methods. Additionally, respondents are usually younger and more liberal than the general public (22). Studies have also shown that respondents who identify as liberal on mTurk may express more left-leaning views in online surveys than they do in practice, which is a potential limitation of this study as the authenticity of the opinions expressed within the survey are difficult to assess (23). Therefore, key informant interviews are a logical follow-up step to gaining a deeper understanding of the preferences and beliefs of any individual veterinary community. Specifically, further investigation is needed to determine if clients would indeed pay the premiums they suggested in our survey. While some studies have shown that consumers are willing to pay 5–23% more for green products (24–27), others have found that, although the majority of consumers indicate environmental concern and awareness, concern does not always translate into actually paying a higher cost for goods and services (24, 28, 29). Additionally, while a large number of veterinary clients were surveyed, the sample size is small compared to the entire United States veterinary clientele. Subsequent studies focusing on specific client populations, the opinions of veterinary practice owners, and weighing sustainability against other factors that influence the choice of a veterinary clinic would help to broaden the scope of our findings.

Although economic considerations are a key driver in implementing change within any business, it is not the only reason for veterinary clinics to address their own ecological footprint. Current (9) and future (8) veterinarians believe that climate change is a significant animal health threat. The veterinary field has a unique opportunity to be leaders in combatting climate change as practice owners and knowledgeable public health professionals through training and implementation of business practices that reduce environmental impacts. Further, through education and outreach about climate change, veterinarians can strengthen their impact on animal health directly and through improved relationships with clients who appreciate the growing importance of climate change in their pet's lives and support a sustainable shift in the veterinary industry. Answering this call will benefit our businesses, clients, patients, and planet.

## Data Availability Statement

The original contributions presented in the study are included in the article/Supplementary Material, further inquiries can be directed to the corresponding author.

## Ethics Statement

The studies involving human participants were reviewed and approved by Colorado State University IRB. Written informed consent for participation was not required for this study in accordance with the national legislation and the institutional requirements.

## Author Contributions

All authors participated in the research project design, conduct, analysis, and preparation of this manuscript.

## Conflict of Interest

The authors declare that the research was conducted in the absence of any commercial or financial relationships that could be construed as a potential conflict of interest.

## Footnotes

^1^Qualtrics®, Qualtrics.com

^2^www.mturk.com

^3^IBM Inc., SPSS Statistics v25.

^4^Microsoft Excel.
