# Supplementary material for: Client Choice May Provide an Economic Incentive for Veterinary Practices to Invest in Sustainable Infrastructure and Climate Change Education
Source: Front Vet Sci. 2021 Jan 18;7:622199. doi: 10.3389/fvets.2020.622199 (PMC7848204; doi:10.3389/fvets.2020.622199)
Supplement: Supplementary file 1 [file Data_Sheet_1.PDF]

Appendix: Survey Questions

Q1 How many total cats and/or dogs do you own currently for which you have received veterinary care **in the past 3 years?**

- 0
- 1
- 2
- 3
- 4
- 5 or more

If '0' was selected for Q1: skip to end of survey and display "Thank you for your interest, but we are only surveying cat and dog owners receiving veterinary care at this time.

Q2 In which state do you live?

- Alaska
- Arizona
- Arkansas
- California
- Colorado
- Connecticut
- Delaware
- Florida
- Georgia
- Hawaii
- Idaho
- Illinois
- Indiana
- Iowa
- Kansas
- Kentucky
- Louisiana
- Maine
- Maryland
- Massachusetts
- Michigan
- Minnesota
- Mississippi
- Missouri
- Montana
- Nebraska
- Nevada
- New Hampshire
- New Jersey
- New Mexico
- New York
- North Carolina
- North Dakota
- Ohio
- Oklahoma
- Oregon
- Pennsylvania

- Rhode Island
- South Carolina
- South Dakota
- Tennessee
- Texas
- Utah
- Vermont
- Virginia
- Washington
- West Virginia
- Wisconsin
- Wyoming
- Other (please list) _________

Q3 With which gender do you identify?

- Male
- Female
- Gender not listed or prefer not to answer

Q4 How old are you?

- 18 to 24 years
- 25 to 34 years
- 35 to 44 years
- 45 to 54 years
- 55 to 64 years
- Age 65 or older

Q5 What is your household income?

- Less than $24,999
- $25,000 to $49,999
- $50,000 to $74,999
- $75,000 to $99,999
- $100,000 to $149,999
- $150,000 to $199,999
- $200,000 or more

Q6 Which of the following best describes the region in which you live?

- Rural (population less than 2,500 people)
- Suburban (population of 2,500-50,000 people)
- Urban (population of over 50,000 people)

Q7 In general, do you think of yourself as:

- Very liberal
- Somewhat liberal
- Moderate, middle of the road
- Somewhat conservative
- Very consesrvative
- Other

Q8 In a typical year, how much money do you spend on veterinary care for your pet(s)?

- $0-50
- $50-99
- $100-299
- $300-599
- $600-999

- $1000 or more

Q9 In the last 3 years, what type of veterinary services have you used? (select all that apply)

- Small animal (dog and cat) veterinary services
- Equine (horse) veterinary services
- Livestock (poultry, sheep, goats, cattle, pigs, etc.) veterinary services
- Exotic animal (birds, small mammals, reptiles, amphibians, fish, etc.) veterinary services
- Other (please list)

Q10 Climate change refers to the idea that the **world's average temperature has been increasing** over the past 150 years, **may be increasing more in the future**, and that the **world's climate is changing as a result.** Do you think that climate change is happening at this time?

- Yes
- No
- I don't know

Q11 How much, if at all, do you think climate change is relevant to your **pet's health**?

- A great deal
- A moderate amount
- Only a little
- Not at all
- I don't know

Q12 In your opinion, climate change is happening on Mars. Please choose 'somewhat agree' if you are paying attention.

- Strongly agree
- Somewhat agree
- Neither agree nor disagree
- Somewhat disagree
- Strongly disagree

Q13 In which of the following ways, if any, do you think veterinary patients are currently being affected by climate change or might be affected in the next 10-20 years?

	Currently			In the next 10-20 years		
	Yes	No	Unsure	Yes	No	Unsure
Declining air quality	○	○	○	○	○	○
Increasing extreme weather events	○	○	○	○	○	○
Increasing vector-borne diseases	○	○	○	○	○	○
Increasing water-associated illnesses/stress	○	○	○	○	○	○
Reduced food safety, quality and security	○	○	○	○	○	○
Increasing heat associated illness/stress	○	○	○	○	○	○

Q14 Are there ways, in addition to those listed above, that you think your pet(s) are currently being affected by climate change, or might be affected in the next 10-20 years? (Fill in answer)

Q15 Please indicate to your level of agreement with the following statements:

	Strongly Agree	Agree	Neither agree nor disagreel	Disagree	Strongly Disagree
I feel that actions I take in my personal and/or professional life can contribute to effective action on climate change	○	○	○	○	○
I consider the sustainability efforts of businesses when deciding where to spend my money on goods and services	○	○	○	○	○
I would like my vet to be knowledgeable about the health impact(s) climate change will have on my animals	○	○	○	○	○
I am interested in the environmental impact of veterinary care	○	○	○	○	○
I would value knowing that my veterinary team had undergone specialized training on the impacts of climate change on the health of animals	○	○	○	○	○
I would value knowing that my veterinary clinic had obtained a certification for their sustainability practices	○	○	○	○	○
I want to be informed about the sustainability efforts made by my veterinary clinic	○	○	○	○	○

Q16 Would you be willing to pay more money for veterinary services at a clinic that has significantly reduced their environmental impact?

- Yes
- No

If 'Yes' was selected for Q16: Continue to Q17
If 'No' was selected for Q16: Skip to Q18

Q17 Approximately how much more would you be willing to pay for veterinary services at a practice that has significantly reduced their environmental impact?

- 1-5%
- 6-10%
- 11-15%
- 16-20%
- 21-25%
- More than 25%

Q18 Which of the following ways, if any, would you like to see veterinary clinics helping to address the issue of climate change?

	Yes	No	I don't know
Providing information and education to clients and the community regarding the health risks of climate change	○	○	○
Purchasing sustainable products for use in the clinic	○	○	○
Maximizing recycling	○	○	○
Using renewable energy	○	○	○
Reducing water waste	○	○	○
Reducing energy usage	○	○	○
Reducing biomedical waste	○	○	○
Recycling of complex (biomedical) products	○	○	○
Offering sustainable pet products for purchase	○	○	○
Using electronic medical records and client communications to reduce paper use	○	○	○
Other, please list or select 'I don't know' if you have no other suggestions	○	○	○
Other, please list or select 'I don't know' if you have no other suggestions	○	○	○
